# Nutrition and Reproductive Health: Sperm versus Erythrocyte Lipidomic Profile and *ω*-3 Intake

**DOI:** 10.1155/2015/670526

**Published:** 2015-10-25

**Authors:** Gabriela Ruth Mendeluk, Mariano Isaac Cohen, Carla Ferreri, Chryssostomos Chatgilialoglu

**Affiliations:** ^1^Laboratory of Male Fertility, Hospital de Clínicas “José de San Martín”, INFIBIOC, Faculty of Pharmacy and Biochemistry, University of Buenos Aires, 5950-800 Buenos Aires, Argentina; ^2^Urology Division, Hospital de Clínicas “José de San Martín”, University of Buenos Aires, 5950-800 Buenos Aires, Argentina; ^3^Consiglio Nazionale delle Ricerche (CNR), Istituto per la Sintesi Organica e la Fotoreattività (ISOF), 40129 Bologna, Italy; ^4^Institute of Nanoscience and Nanotechnology, National Center of Scientific Research “Demokritos”, Agia Paraskevi, 15310 Athens, Greece

## Abstract

Fatty acid analyses of sperm and erythrocyte cell membrane phospholipids in idiopathic infertile patients evidenced that erythrocyte contents of EPA, DHA, omega-6–omega-3 ratio and arachidonic acid provide a mathematical correspondence for the prediction of EPA level in sperm cells. The erythrocyte lipidomic profile of patients was significantly altered, with signatures of typical Western pattern dietary habits and no fish intake. A supplementation with nutritional levels of EPA and DHA and antioxidants was then performed for 3 months, with the follow-up of both erythrocyte and sperm cell membranes composition as well as conventional sperm parameters. Some significant changes were found in the lipidomic membrane profile of erythrocyte but not in sperm cells, which correspondently did not show significant parameter ameliorations. This is the first report indicating that membrane lipids of different tissues do not equally metabolize the fatty acid elements upon supplementation. Molecular diagnostic tools are necessary to understand the cell metabolic turnover and monitor the success of nutraceuticals for personalized treatments.

## 1. Introduction

Decreasing the number of men affected by infertility has become a top priority for many health organizations, including Healthy People 2020. Modifiable lifestyles should then be considered before deciding high complexity assisted reproduction techniques clearly bypassing the natural barriers of human development. Age when starting a family, nutrition, weight management, exercise, psychological stress, cigarette smoking, recreational drugs use, medications, alcohol use, caffeine consumption, environmental and occupation exposure, preventive care, clothing choices, hot water, and lubricants are among them [1].

Evidence suggests that male fertility decreases by men being either overweight or underweight (as defined by body mass index [BMI] > 25 kg/m^2^ and BMI < 20 kg/m^2^, resp.) [2]. Healthy diet and regular exercise are therefore both recommended to maintain BMI between 20 and 25 kg/m^2^ [3]. While considering nutrition, correlation among obesity, metabolic syndrome (MS), and reproductive axis was observed [4]. Excess adipose tissue results in increased conversion of testosterone to estradiol, which may lead to secondary hypogonadism through reproductive axis suppression. The benefits of low-fat intake in male fertility have recently been reported [5].

In a preliminary cross-sectional study, high intake of saturated fats was negatively related to sperm concentration whereas higher intake of omega-3 fats was positively related to sperm morphology [6]. On the other hand, the negative impact of nutritional deficiencies on semen quality after bariatric surgery was reported [7–9]. Moreover, an adequate intake of certain fatty acids is necessary for an adequate spermatogenesis [10]. In a recent study, it was found that 20- and 22-carbon fatty acids are present in semen in much larger quantities than thus far reported in any other mammalian tissue [11]. The first mechanism by which *ω*-3 and *ω*-6 PUFAs affect spermatogenesis is by the incorporation into spermatozoa cell membrane. *ω*-3 and *ω*-6 PUFAs are structural components of cell membranes [12], and the lipid bilayer properties of fluidity and permeability are maintained by the presence of these PUFAs [13]. Indeed, the successful fertilization of spermatozoa depends on the lipids of the spermatozoa membrane [14]. It has been recently been demonstrated that walnuts added to a Western-style diet improved sperm vitality, motility, and morphology [15]. There is no doubt that diet is a modifiable factor that could impact male fertility [16].

The aim of this study was to evaluate erythrocyte and sperm cell membrane fatty acids before and after 3 months of supplementation with omega-3 in parallel with sperm quality parameters in a group of ten “idiopathic infertile” patients, examined by the andrologist and nutritionist staff.

## 2. Material and Methods

### 2.1. Study Design

Evaluation of erythrocyte and sperm lipidomic profile from infertile patients was carried out at the beginning of the study while being evaluated by andrologists and nutritionists. In case of fatty acid deficiencies of erythrocyte composition determined in comparison with healthy interval values reported in literature [21], therapeutic supplementation was carried out for three months. Changes after treatment were analysed.

### 2.2. Patients

A total of 10 “idiopathic infertile” patients were recruited. Varicocele, infection, or genetic diseases were discarded. Demographic, clinical, and nutritional data were recorded in an interview taken by the physician. Height, weight, nationality, smoking addiction, physical activities, and medical anamnesis were registered.

The present study was conducted according to the guidelines laid down in the Declaration of Helsinki and was approved by the Institutional Review Board of The University Clinical Hospital “José de San Martin” (Buenos Aires, Argentina). All the participants received information on the project and gave written informed consent to be included.

### 2.3. Dietary Measures

Usual diet at baseline was determined using a self-administered nutritional questionnaire guided by trained nutritionists. The frequency per week for each food type and the quantity per portion were registered, underlying dietary patterns.

### 2.4. Lipidomic Profile

Blood samples, obtained from the patients, were collected in K2-EDTA vacutainers (Becton, Dickinson and Company, Franklin Lakes, NJ, USA). The erythrocyte fatty acid membrane profile analysis was carried out as previously described, using the erythrocyte membrane pellet obtained by standard methods [18].

Briefly, the phospholipid fraction of the membrane pellet was treated with 0.5 M KOH/MeOH for 10 min at room temperature, and the corresponding fatty acid methyl esters (FAMEs) were formed and extracted with* n*-hexane. Fatty acid methyl esters were analyzed by GC (Agilent 6850, Milan) equipped with a 60 m × 0.25 mm × 0.25 *μ*m (50%-cyanopropyl)-methylpolysiloxane column (DB23, Agilent, USA) and a flame ionization detector (FID) with the following oven program: temperature started from 165°C, held for 3 min, followed by an increase of 1°C/min up to 195°C, held for 40 min, followed by a second increase of 10°C/min up to 240°C, and held for 10 min. A constant pressure mode (29 psi) was chosen with helium as carrier gas. Methyl esters were identified by comparison with the retention times of authentic samples. Geometrical TFAs (trans fatty acids) were recognized by comparison with standard references obtained by synthesis, as already described [17]. The amounts of the individual FAME were calculated as a percentage of the total measured FAMEs and their standard deviations calculated in Excel.

For sperm lipidomic profile of the semen, seminal plasma was discarded and the cells were washed in PBS (pH = 7.8). One mL of buffer containing at least 6 × 10^6^ spermatozoa was used to isolate membrane PL according to the described procedure. Two repetitions for the same analysis were performed. Two repetitions for the analysis of the same sample were performed.

### 2.5. Nutraceutical Supplementation

Commercial soft-gel capsules containing *ω*-3 fatty acid-based formulas from fish and algal oils were used. Patients visited the physician three times during the study: at baseline and one month and two months later. In each visit the andrologist gave them the capsules for the next month and asked them if they had any problem with the intake. He verified in all the cases that it was correctly performed, receiving the empty blister each month.

#### 2.5.1. Nutritional Values and Analysis for Capsule

A commercial soft-gel capsule containing *ω*-3 fatty acids from fish oil was used (Lipinutragen srl, Bologna, Italy). The main components and quantities of each capsule are described as follows: 5.31 Kcal; KJ 21.95; protein 0.17 g; 0.04 g carbohydrates; fat 0.510 g of which fatty acids are such as 0.040 g saturated; 0.042 g monounsaturated; and 0.323 g polyunsaturated of which EPA (eicosapentaenoic acid) 0.103 g, DHA (docosahexaenoic acid) 0.070 g, and ALA (alfa-linolenic acid) 0.070 mg; vitamin C 90.00 mg (112.5% RDA); vitamin E 10.00 mg (*α*TE; 83.3% RDA); astaxanthin 2.00 mg; and L-*α*-glyceryl phosphoryl choline 90 mg.

#### 2.5.2. Nutritional Values and Analysis for Capsule

A commercial soft-gel capsule containing DHA from algal oil was used. The main components and quantities of each capsule are described as follows: 5.118 Kcal; KJ 21.14; protein 0.18 g; carbohydrates 0.08 g; fat 0.450 g of which fatty acids are such as 0.053 g saturated; 0.037 g monounsaturated; 0.314 g polyunsaturated fatty acids of which DHA is 0.200 g; vitamin C 90.00 mg (112.5% RDA); vitamin E 10.00 mg (*α*TE; 83.3% RDA); astaxanthin 2.00 mg; and L-*α*-glyceryl phosphoryl choline 100 mg.

### 2.6. Sperm Assays

Conventional sperm assay was performed according to WHO criteria (2010) [20]. Samples were obtained by masturbation after a period of 3 to 5 days of sexual abstinence. Subjective motility was evaluated by expert technicians. Sperm motility was classified as progressive motility (PR), nonprogressive motility (NP), and immotility (IM). Sperm vitality was evaluated using eosin Y; the sample was stained with 0.5% eosin Y in 0.9% aqueous sodium chloride solution; bright red (dead) and uncolored (living) cells were scored by light microscope. Finally, semen smears were Papanicolaou stained and morphology was assessed using Kruger's strict criteria. Conventional sperm parameters were also expressed in absolute values as recommended by WHO [20].

A Sperm Class Analyzer CASA system (SCA Microptic SL, Barcelona, Spain) was employed to assess kinetic parameters and sperm count. The basic components of the system are a bright field microscope with phase contrast Ph- (negative phase contrast) microscopy to visualize the sample (Nikon E- 200, Japan) lens magnification: 10x, a digital camera to capture images (Basler A312 Inc., Vision-Technology, Germany), and a computer with SCA software installed. Slides with samples were laid on a thermostatic plate at 37°C. A minimum of 400 sperm cell tracks were captured and 25 digitized images per second were analyzed for each sample. The assays were conducted in accordance with instrument's standardization and validation, by using a Leja chamber 10 (10 *μ*m in depth) [22]. A qualified operator validated each analyzed image. The improved Neubauer haemocytometer was used only in cases of very low count. Appropriate dilutions were made in Mac Comber fixative (formaldehyde: 1 mg/mL, NaHCO3: 5 g made up to 100 mL with purified water).

### 2.7. Statistic

Evaluation results after supplementation were based on Wilcoxon signed ranks test. Multiple linear regression was employed to predict sperm fatty acid profile (Software: Infostat-Universidad Nacional de Córdoba; 2014 version). *p* values less than 0.05 were considered statistically significant.

## 3. Results

### 3.1. Patient's Data

Demographic data are shown in Table 1. Only two patients showed relevant clinical data, one suffering asthma and the other parathyroid cancer. While analyzing the food intake questionnaire, interesting data were recorded; the main data is that neither of the participants consumed fish; their diet was based on red meat and carbohydrates, that is, the typical “Western diet.” Their infertility history ranged from secondary infertility to 6 years of primary infertility.

### 3.2. Red Blood Cell Membrane Lipidomic Profile

In erythrocytes membranes, it was possible to monitor the fatty acid residues of membrane phospholipids, and we were interested in seeing the changes of the EPA and DHA levels in the patients while comparing baseline and postsupplementation profile, with variation of the fatty acid levels after supplementation.

The values were reported as relative percentages of a cohort of 12 fatty acids chosen as the most significant components of the erythrocyte membrane fatty acids [18, 21]. The percentages were obtained from the gas chromatographic analysis (GC) of fatty acid methyl esters derived from the membrane phospholipids, following previously published procedures [17]. GC is the gold standard for the fatty acid analysis and the relative percentages in erythrocyte membranes were used in other widely used lipid biomarker evaluations, such as the *ω*-3 cardiovascular risk index [19].

The comparison of the found values in the patient cohort could also be made with known interval values registered for the Italian population and integrated with literature data reported for erythrocyte membranes of healthy subjects [21].

At the beginning of the study, all the patients had low levels of EPA and DHA in red blood cells membranes and increased values of the saturated fatty acid/monounsaturated fatty acid and *ω*6/*ω*3 ratios (Table 2). Eight of the 10 participants had a high cardiovascular risk index, measured in erythrocyte membranes as % eicosapentaenoic + docosahexaenoic acid (0–4%) [19].

From the membrane lipid profile obtained in the patient cohort, it was possible to envisage a decreased functionality due to poor polyunsaturated components such as *ω*-3 fatty acids. Further evaluation of the erythrocyte functionality will be matter of specific studies. It is worth noting that the fish intake is known to influence the presence of the *ω*-3 long chain fatty acids and give health benefits [23], and the food questionnaire of these patients showed no fish consumption. Besides being involved as disease risk, the *ω*-3 fatty acids in membranes are recognized as essential elements for the correct functionality [24]. There is no doubt on the need of recovering the membrane status of these patients; therefore a supplementation was assigned to follow for 3 months.

After supplementation of 1 capsule of omega-3-based nutraceutical and 1 capsule of DHA-based nutraceutical per day to the patients (see Materials and Methods), the erythrocyte membrane profiles were obtained. The effects of supplementation were clearly visible. They were statistically significant for EPA (*p* < 0.01) and DHA (*p* < 0.03). In particular, arachidonic acid levels in the erythrocyte membranes decreased after supplementation (*p* < 0.009). This could be indicated as favourable remodelling of the erythrocyte membranes due to the *ω*-3 supplementation, with exchange of membrane lipids with the fatty acid pool enriched with the *ω*-3 supplementation, so that less inflammatory status can be obtained from a better *ω*-6/*ω*-3 balance. Considering one patient who exited from the study, the risk of cardiovascular disease after treatment decreased to the intermediate range (4–8%) in 8 out of the 9 studied patients.

### 3.3. Sperm

Only two out of the studied patients were asthenospermic, while the rest were severely comprised as being oligoasthenoteratospermic. In sperm cells, the fatty acids were also evaluated before and after supplementation.  Table 4 reports the sperm cell lipidomic profile with the full recognition of all fatty acids, expressed as relative percentages of the total fatty acid found in the samples. It is important to note that in Table 4 the fatty acids were recognized with the library of cis- and trans fatty acid, as described elsewhere [17, 25]. It is remarkable that while conventional sperm parameters (Table 3) were recorded after treatment of 3 months with the omega-3 formulations and no changes were detected, sperm lipidomic profile after supplementation also did not show significant changes. Due to the low sperm count found in most of the patients, sperm kinetics could only be evaluated in four of them. Slight differences could be envisaged in average path and curvilinear velocity after treatment (Table 3).

EPA, DHA, the *ω*6/*ω*3 ratio, and arachidonic acid in erythrocytes were good predictors of EPA in sperm (*R* ^2^: 0.975) with *β*_coefficient_: *β*_ARA_ = 0.23 (*p*-value = 0.020); *β*_EPA_ = −1.474 (*p* value = 0.020); *β*_DHA_ = 0.871 (*p* value = 0.004); and *β* *ω*6/*ω*3 = 0.189 (*p* value = 0.014) at the beginning of the study, the prediction equation being

(1) EPAsperm=−7.8+0.23arachidonic acidery−1.474EPAery+0.871DHAery+0.189ω6ω3ery.

After the supplementation, the correspondence between sperm and erythrocyte was completely lost.

## 4. Discussion

The fatty acid analysis and lipidomic profiles of erythrocytes and sperm cells from infertile patients were performed following published protocols [14, 17]. Nutraceutical treatment was effected with 1 capsule of *ω*-3 and 1 capsule of DHA per day for 3 months.

After the supplementation, the level of the *ω*-3 fatty acids in plasma can be obviously found more elevated; however we were interested in following the incorporation in phospholipid structures that can be much less efficient. Phospholipids of red blood cells are of great value for their fatty acid asset as multifaceted expression of* de novo* biosynthesis, nutrient uptake, and exchange for efficient tissue maintenance. The mean erythrocyte lifetime of 120 days renders the membrane lipidome analysis strongly related to the stabilized dietary habits and to the individual metabolic status. The diet evolved with a ratio of *ω*-6/*ω*-3 essential fatty acids of approximately 3/1-4/1 along the centuries of human development, whereas in the last century with industrialized diets (denominated “Western diets”) the ratio reached 20–30/1 [24]. The change of *ω*-6/*ω*-3 ratio has been correlated with the pathogenesis of many diseases including cardiovascular disease, cancer, and inflammatory and autoimmune diseases. Despite the strong correlation between fatty acids and health conditions, a very scarce application of lipidomic tools has been observed for monitoring dietary intakes of fatty acids and their consequent molecular contribution. The new field of nutrilipidomics indicates the strategy of lipidomic molecular diagnostics to envisage the nutritional elements needed to be supplemented to the individual in a personalized manner [18, 21]. This strategy takes into account two main factors: (i) the target of a lipid intervention, addressing membranes as the most important functional compartment of fatty acids in living organisms; (ii) the personalization of the type and dose of fatty acids to be used and the importance to use cocktail of fatty acids with molecules able to control free radical and oxidation processes during biodistribution. The role of multicomponent supplementation containing fatty acids in order to enhance the bioavailability of the supplementation will be matter of further studies.

Our results were followed up by clinicians who evidenced that the patients regularly took the nutraceuticals and declared to have improved their general status. The lipid composition of the sperm cell membrane has been shown to exert a significant effect upon the functional quality of spermatozoa. Compared with normozoospermic samples, asthenozoospermic samples showed lower levels of PUFA and higher amount of saturated fatty acids [26]. In accordance with data reported by Lenzi et al. [14], we found that palmitic and stearic acids were the most representative saturated fatty acids in whole spermatozoa, oleic acid was the most frequent monounsaturated one, the essential fatty acid, linoleic acid, was the main *ω*-6 fatty acid, and DHA was the most representative long chain PUFA *ω*-3 of mature sperm cells. In spite of this general feature, our data on DHA were much lower than the data reported by the authors, being comparable to those found by the same group in immature germ cells. The fact that the patients gave the indication of low fish intake in the diet can be explicative of our findings. A higher DHA content was associated with better sperm morphology and function. On the other hand, it is well known that polyunsaturated fatty acids are particularly susceptible to peroxidation damage by free radicals. Although reactive oxygen species (ROS) play significant role for physiological sperm function, when the production of potentially destructive ROS exceeds the natural antioxidant defences, oxidative stress is connected with cell damage. Moreover, oxidative stress at the level of the testicular microenvironment may result in decreased spermatogenesis and sperm damage. Total antioxidant capacity in seminal plasma was directly related to PUFA, omega-3, and DHA. Detrimental effects of lipid peroxidation should decrease sperm quality and be responsible for fertility problems, so we believe that the higher contents of DHA in good sperm samples are dependent not only on nutrition but also on the overall antioxidant capacity of each person. We did not perform any specific measurement of oxidative stress nor evaluated ROS production in the gamete and neither did we determine the lipid profile in seminal plasma that was not the target of this study, focused on the sperm. The spermatozoa are in tight equilibrium with seminal plasma and are finally the responsible male cell of fertilization outcome.

The supplementation of EPA and DHA was at a dosage that simulated the intake of *ω*-3 fatty acids from dietary sources. Indeed, several reports regarding *ω*-3 fatty acid supplementation made use of higher dosage, therefore not simulating dietary intakes and finding correlation between the supplementation and the dosage of *ω*-3 fatty acids in the body [27]. In our case, the findings that 3 months of supplementation of nutritional dosages of *ω*-3 fatty acids is successful for erythrocyte membranes are important for further studies on the “effective” dosages to be used in nutraceutical interventions that reach sperm cells.

We could verify that 3 months of supplementation did not change conventional sperm parameters in a significant manner. Data on sperm kinetics, although few, are promising to envisage slight differences in sperm function; they should be further studied. Our results give important and first evidence that there are quite different distribution rates of supplemented fatty acid in the membrane lipids of different tissues, comparing the changes recorded in erythrocytes and sperm cells of the same patient. Therefore, molecular diagnostic tools that are of great help in understanding clinical outcomes can monitor the success of supplementations, as well as of dietary treatments. As it could be expected, the statistical prediction equation could not be applied to the erythrocyte and sperm values after supplementation.

The *ω*6/*ω*3 ratio was much higher in our patients than the one found in oligoasthenospermic samples by Aksoy et al. [28]. A balanced *ω*6/*ω*3 ratio proved to be necessary in male rat reproduction highlighting the necessity to determine an appropriate *ω*6/*ω*3 ratio in man in the future [29].

Spermatogenesis is a very complex, highly organized and regulated process that takes place in the seminiferous epithelium of testis tubules and involves three major fundamental biological processes: the renewal of stem cells and the production and expansion of progenitor cells (mitosis); the reduction, by one-half, of the number of chromosomes in each progenitor cell (meiosis); and the unique differentiation of haploid cells (spermiogenesis). In our experience, three months of nutraceutical supplementation was not sufficient to remodel sperm membrane lipidomics, at least in these patients having also severe alteration in their red blood cell membrane lipidomic profile. Our study encourages longer trials with omega-3 supplementation to allow sperm lipidomic to adjust and then evaluate the treatment in regard to the irreversibility of some teratospermic cases.

It would be interesting to pursue a multicentric survey of lipidomic profiles in order to expand the data from the initial collection of data performed with the Italian population [18, 21] and also compare geographically different patient cohorts with the average of healthy controls. As a matter of fact, the profound deficit of *ω*-3 EPA and DHA in the patients with increased arachidonic acid presence can create proinflammatory conditions, which are responsible for impaired functionality of cells. The reduced levels of *ω*-3 express the fish intakes [30], which is in fact absent in the cohort and nowadays is remarked as responsible for health impairment [23, 31]. Overall, the *ω*-3 contribution to membrane asset and functionality is widely recognized [32]; therefore the clinical outcomes cannot be established without taking into account the personalized fatty acid levels and needs.

We agree with Tavilani et al. [33] who stated that lipid content is regulated locally within the male reproductive tract. To our knowledge this is the first report on a mathematic equation that could predict EPA in sperm by EPA, DHA, the ratio *ω*-6/*ω*-3, as compared with optimal values found in literature [18, 21], and arachidonic acid in erythrocyte membranes, thus reflecting the real impact of nutrition on individual male reproductive health in regard to fatty acid intake. We interpret the idea that the equilibrium installed between sperm and red cell lipidomic profile in relation to diet is disrupted by treatment. A first hypothesis is that administration of the capsules for a longer period would have redounded in a new equilibrium favouring spermatogenesis. More generally speaking, the approach of molecular diagnostics together with the parallel analysis of erythrocyte and sperm cell membranes can be suggested as diagnostic approach helping the clinical observation of diseases such as, in our particular scenario, the “male fertility.”

## Figures and Tables

**Table 1:** Demographic data.

Patient	Age	Height (cm)	Weight (kg)	Body mass index (kg/m^2^)	Smoker	Job type	Physical activity	Sport
1	33	170	71	24.6	Ex	Architect	Light	No
2	31	177	80	25.5	Ex	Builder	Light	No
3	37	178	83	26.2	No	Driver	No	No
4	32	172	75	25.4	Yes	Teacher	Medium	Yes
5	44	180	95	29.3	No	Mechanic	Light	No
6	35	171	84	28.7	Yes	Gardener	Strong	Yes
7	32	170	65	22.5	No	Stonemason	Light	No
8	32	169	92	32.2	Yes	Seller	No	No
9	37	164	89.4	32.2	Yes	Employee	Light	No
10	30	185	120	35.1	Ex	Operator	Light	No

Data recorded after the clinical interview: BMI categories: underweight = 18.5; normal weight = 18.5–24.9; overweight = 25–29.9; obesity = BMI of 30 or greater.

**Table 2:** Fatty acid profile in erythrocyte membranes.

FAME^a^	Subjects before supplementation (*n* = 10; % rel)	Subjects after supplementation (*n* = 9; % rel)	Optimal interval values (% rel)^b^	*p* value
16:0	26.8 (24.3; 29.8)	27.3 (24.1; 29)	17–27	0.8326
18:0	20.7 (19.1; 21.4)	19.5 (16.9; 21.5)	13–20	0.0294
9cis-16:1	0.4 (0.2; 0.6)	0.6 (0.4; 0.7)	0.2–0.5	0.04
9cis-18:1	15.8 (14.2; 18.3)	16.0 (14.3; 17.7)	9–18	0.2138
11cis-18:1	1.4 (1.1; 1.7)	1.3 (1; 1.6)	0.7–1.3	0.4072
18:2 (omega-6, LA)	10.3 (9.5; 14.3)	12.3 (10.0; 14.7)	9–16	0.0044
20:3 (omega-6, GLA)	2.0 (1.4; 3.4)	2.3 (1.3; 3.3)	1.9–2.4	0.863
20:4 (omega-6, ARA)	18.1 (16.6; 19.1)	15.9 (14.0; 19.2)	13–17	0.0092
20:5 (omega-3, EPA)	0.4 (0.2; 0.5)	0.9 (0.6; 1.2)	0.5–0.9	0.0112
22:6 (omega-3, DHA)	3.3 (2.2; 4.5)	4.3 (2.7; 6.3)	5–7	0.0366
Trans-18:1^c^	0.2 (0.1; 0.3)	0.1 (0; 0.2)	0.1–0.3	0.0306
Total SFA	47.5 (45.0; 50.7)	46.9 (42.6; 48.5)	30–45	0.1636
Total MUFA	17.5 (16.1; 20.6)	18.1 (15.9; 19.7)	13–23	0.2856
Total PUFA	34.2 (32.4; 38.2)	35.5 (31.5; 39.8)	28–39	0.3868
SFA/MUFA	2.7 (2.3; 3.1)	2.5 (2.4; 3.0)	1.7–2	0.0196
Omega-6/omega-3	8.4 (6.2; 13.7)	5.7 ± (4.5; 9.4)	3.5–5.5	0.01
CV risk index^d^	3.6 (2.6; 4.8)	5.2 (3.4; 7.2)	0–4%, high risk 4–8%, intermediate risk >8%, minimal risk	0.0098

The values of the fatty acids are reported as relative percentage (% rel) of the total of 12 fatty acids chosen as representative components of the erythrocyte membrane fatty acids GC analysis as reported in [17]. The values were given as median (min; max). *n* is the number of subjects. ^a^FAME (fatty acid methyl ester) was determined performing membrane phospholipid extraction, derivatization, and GC analysis as described. The identification of the peaks was performed using authentic samples as described [17, 18]. ^b^The optimal interval values are reported from [17], as found in a survey of healthy controls reported in the scientific literature and compared with a group of 2500 analyses obtained from the Italian population].  ^c^9-trans-18:1 is considered. ^d^As described in [19], *p* values less than 0.05 were considered statistically significant.

**Table 3:** Sperm parameters.

Sperm parameter	Before treatment (*n* = 10)	After treatment (*n* = 9)	*p* value
Semen volume (mL)	3.0 (1.7; 7.5)	3.5 (1.2; 4.5)	0.52
Sperm concentration (×10^6^/mL)	1.5 (0.05; 53.6)	1.1 (0; 64.3)	0.09
Total sperm count (×10^6^/ejaculate)	3.28 (0.09; 214.4)	4.95 (0; 211.75)	0.85
Progressive motility (%)	4.5 (0; 36.9)	3 (0; 20)	0.32
Total progressive motility (×10^6^/ejaculate)	0.3 (0; 24.45)	0.18 (0; 11.63)	0.60
Normal morphology (%)	2 (0; 14)	0 (0; 11)	0.72
Total normal morphology (×10^6^/ejaculate)	0 (0; 30.02)	0 (0; 23.29)	0.49
Vitality (%)	55 (0; 89)	66 (0; 80)	0.37
Total vitality (×10^6^/ejaculate)	0.63 (0; 190.82)	0.8 (0; 156.7)	0.66
Average path velocity (*μ*m/sec)	33.65 (10.5; 66.8)	20.05 (7.6; 43.3)	0.04
Straight line velocity (*μ*m/sec)	27.2 (5.8; 59.1)	16.7 (6.3; 36.2)	0.16
Curvilinear velocity (*μ*m/sec)	41.6 (19.6; 83.7)	27.5 (9.4; 59.2)	0.04

The values are expressed as median (min; max). Sperm assays were performed according to WHO criteria (2010) [20] (*n* = 9). The kinetic parameters were determined with CASA system (SCA Microptic) (*n* = 4). *p* values less than 0.05 were considered statistically significant.

**Table 4:** Fatty acid profile in sperm cells isolated from the human.

FAME^a^	Samples before supplementation (*n* = 8; % rel)	Samples after supplementation (*n* = 8; % rel)	*p* value
14:0	1.4 (0.83; 1.84)	1.41 (0.76; 2.55)	0.61
16:0	33.3 (30.23; 40.78)	33.79 (24.12; 35.41)	0.25
17:0	3.21 (1.28; 3.99)	2.08 (1.25; 2.82)	0.12
18:0	12.67 (7.55; 16.47)	10.33 (8.73; 27.07)	0.75
20:0	2.5 (1.02; 3.05)	1.54 (1.11; 2.63)	0.48
22:0	7.67 (2.05; 10.12)	4.53 (1.78; 12.54)	0.76
TOT. SFA	58.03 (52.06; 64.22)	55.01 (38.72; 66.74)	0.19
6c 16:1	1.38 (1.0; 1.78)	1.9 (0.65; 3.98)	>0.99
9c 16:1	3.99 (2.63; 6.31)	3.68 (1.72; 18.15)	>0.99
9c 18:1	9.85 (6.56; 12.85)	8.52 (7.2; 15.15)	0.44
11c 18:1	6.96 (3.77; 9.72)	4.79 (2.05; 19.27)	0.87
20:1	3.33 (0.57; 6.79)	3.83 (1.15; 11.87)	0.29
22:1	1.25 (0.33; 2.14)	0.88 (0.26; 1.56)	0.12
TOT. MUFA	27.48 (18.96; 30.67)	26.5 (20.29; 52.94)	0.54
SFA/MUFA	2.17 (1.84; 2.81)	2.17 (0.73; 3.01)	0.4
18:2	4.21 (3.32; 5.56)	5.0 (2.5; 6.19)	0.37
20:2	0.66 (0.16; 1.48)	0.36 (0.26; 1.25)	0.48
20:3	1.2 (0.37; 2.44)	1.21 (0.65; 2.54)	0.91
20:4 (arachidonic acid)	1.54 (0.91; 2.47)	1.23 (0.63; 3.21)	0.41
TOT. *ω*6	7.54 (6.77; 9.71)	8.12 (5.0; 10.27)	0.71
20:5 (EPA)	0.46 (0.16; 1.2)	0.31 (0.17; 0.59)	0.34
22:5	0.95 (0.44; 2.41)	0.62 (0.35; 3.72)	0.15
22:6 (DHA)	3.02 (1.46; 19.47)	3.5 (1.41; 10.4)	0.71
TOT. *ω*3	4.59 (3.02; 22.06)	4.45 (1.93; 11.79)	0.78
*ω*6/*ω*3	1.75 (0.34; 2.57)	1.59 (0.62; 3.3)	0.46
Trans fatty acid (9t-18:1)	0.18 (0; 0.65)	0.12 (0; 0.26)	0.24

The values of the fatty acids are reported as relative percentage (% rel) of the fatty acid peak areas detected in the GC analysis (>98% recognized peaks). The values were given as median (min; max). *n* is the number of samples. ^a^FAME (fatty acid methyl ester) was determined performing membrane lipid extraction, derivatization, and GC analysis as described [17, 18]. *p* values less than 0.05 were considered statistically significant.
